# Supplementary material for: Content-rich biological network constructed by mining PubMed abstracts
Source: BMC Bioinformatics. 2004 Oct 8;5:147. doi: 10.1186/1471-2105-5-147 (PMC528731; doi:10.1186/1471-2105-5-147)
Supplement: Additional File 2 — The original results of the above study (non-essential files are deleted to keep the file size under the limit set by BMC bioinformatics). [file 1471-2105-5-147-S2.bz2 › chilibotAdditionalFile2/dip10/228ID7954478E745/html/CTNNB1_CDH1.html]

 


 **CTNNB1** and **CDH1** 
  
Found 952 abstracts in PubMed, retrieved 10.  
 

 What does Google say? 
 PDF only 
| .edu only 

---

**Interactive relationship** (e.g. stimulation, inhibition, etc)

**Inhibitory relationship**- Reduced expression of E cadherin  [ **CDH1** ] , which interacts with beta catenin  [ **CTNNB1** ]  at the membrane, was observed in some tumors, but this did not correlate with Catnb mutation.  Ref: 12878043 Toxicol Appl Pharmacol, 2003
- In conclusion our results suggested that beta catenin  [ **CTNNB1** ]  alteration might be a minor contributor to the development of gallbladder carcinomas through abnormal Wnt wingless signalling decreased membranous expression of beta catenin  [ **CTNNB1** ]  might be correlated to carcinoma progression through loss of cell adhesive function in E cadherin  [ **CDH1** ]  catenin  [ **CTNNB1** ]  fashion.  Ref: 12866584 J Exp Clin Cancer Res, 2003
**Neutral relationship**- As shown by immunoprecipitation, the re expressed E cadherin  [ **CDH1** ]  was able to sequestrate one part of free cytoplasmic beta catenin  [ **CTNNB1** ]  in BZR cells.  Ref: 12875984 Am J Pathol, 2003

**Non-interactive relationship** (e.g. studied together, co-existance, homology, etc.)

- Expression of E cadherin  [ **CDH1** ] , alpha catenin  [ **CTNNB1** ] , and beta catenin  [ **CTNNB1** ]  in the process of lymph node metastasis in oral squamous cell carcinoma.  Ref: 12888830 Br J Cancer, 2003
- Significantly greater reduction in expression levels of E cadherin  [ **CDH1** ] , alpha catenin  [ **CTNNB1** ]  and beta catenin  [ **CTNNB1** ]  was found in the metastatic group n=64 compared to the nonmetastatic group n=95 P=0.00.  Ref: 12888830 Br J Cancer, 2003
- Expression of potential beta catenin  [ **CTNNB1** ]  targets, cyclin D1, c Jun, c Myc, E cadherin  [ **CDH1** ] , and EGFR in chemically induced hepatocellular neoplasms from B6C3F1 mice.  Ref: 12878043 Toxicol Appl Pharmacol, 2003
- Western blotting was used to determine polypeptide size and expression of E cadherin  [ **CDH1** ]  and beta catenin  [ **CTNNB1** ]  in Wilms s tumours compared with normal kidney.  Ref: 12890743 Mol Pathol, 2003
- We investigated immunohistochemical expression of E cadherin  [ **CDH1** ] , alpha catenin  [ **CTNNB1** ]  and beta catenin  [ **CTNNB1** ]  in 159 tissue samples from patients with oral squamous cell carcinoma and examined the correlation between their expressions and the presence of regional lymph node metastasis.  Ref: 12888830 Br J Cancer, 2003
- These data demonstrate a direct role of E cadherin  [ **CDH1** ]  catenin  [ **CTNNB1** ]  complex organization in the regulation of MMPs and suggest an implication of this regulation in the expression of an invasive phenotype by bronchial tumor cells.  Ref: 12875984 Am J Pathol, 2003
- Distribution of cell cell adhesion molecules of E cadherin  [ **CDH1** ]  EC and beta catenin  [ **CTNNB1** ]  BC infiltrating breast carcinoma BC and markers of epithelium, basal membrane has been studied by immunofluorescence in 50 infiltrating BC tissue samples including 17 ductal carcinomas, 23 lobular carcinomas and 10 combined carcinomas.  Ref: 12879601 Arkh PatolArkh Patol, 2003
- The disorganization of E cadherin  [ **CDH1** ]  catenin  [ **CTNNB1** ]  complexes and the overexpression of matrix metalloproteinases MMPs are frequently involved in the capacity of epithelial cells to acquire an invasive phenotype.  Ref: 12875984 Am J Pathol, 2003
- We have studied the effects of the organophosphorous pesticide malathion on cell viability, actin cytoskeleton, cell adhesion complex E cadherin  [ **CDH1** ]  beta catenin  [ **CTNNB1** ] , and Rho and Rac1 GTPases from the human mammary carcinoma cell line MCF 7.  Ref: 12888906 Int J Oncol, 2003
- AIMS To evaluate E cadherin  [ **CDH1** ] , beta catenin  [ **CTNNB1** ] , gamma catenin  [ **CTNNB1** ] , and ezrin expression and its relation to histopathological features of primary and metastatic Wilms s tumours.  Ref: 12890743 Mol Pathol, 2003
- BACKGROUND The E cadherin  [ **CDH1** ]  catenin  [ **CTNNB1** ]  adhesion complex is crucial for intercellular adhesiveness and maintenance of tissue architecture.  Ref: 12890743 Mol Pathol, 2003
- Immunocharacterization with beta catenin  [ **CTNNB1** ] , E cadherin  [ **CDH1** ] , cytokeratins AE1 AE3, epithelial membrane antigen, S 100 protein, smooth muscle actin, and vimentin was also performed.  Ref: 12883237 Am J Surg Pathol, 2003
- CONCLUSIONS Compared with primary tumours, metastatic tumours showed lower expression of E cadherin  [ **CDH1** ]  and gamma catenin  [ **CTNNB1** ] , with nuclear staining for beta catenin  [ **CTNNB1** ] .  Ref: 12890743 Mol Pathol, 2003
- In this study, we screened liver tumors induced by 2 amino dimethylimidazo f quinoline MeIQ in CDF 1 mice for beta catenin  [ **CTNNB1** ]  and APC mutations and other genetic alterations shown to occur in human hepatocellular carcinomas HCC, including mutations in the p53 and H ras genes, c myc amplification and E cadherin  [ **CDH1** ]  promoter methylation.  Ref: 12893427 Cancer Lett, 2003
- These changes were related to a decrease in the expression of the adhesion molecules, E cadherin  [ **CDH1** ]  and beta catenin  [ **CTNNB1** ] , and to the distribution and reactivity of actin microfilaments to TRITC phalloidin.  Ref: 12888906 Int J Oncol, 2003
- The decrease of beta catenin  [ **CTNNB1** ]  transcriptional activity in E cadherin  [ **CDH1** ]  transfected clones was demonstrated using the TOP FLASH reporter construct.  Ref: 12875984 Am J Pathol, 2003
- METHODS Immunohistochemistry was used to determine the expression and cellular distribution of E cadherin  [ **CDH1** ] , beta catenin  [ **CTNNB1** ] , gamma catenin  [ **CTNNB1** ] , and ezrin in primary and metastatic Wilms s tumours.  Ref: 12890743 Mol Pathol, 2003
- These data suggest that evaluation of the immunohistochemical expression of E cadherin  [ **CDH1** ] , alpha catenin  [ **CTNNB1** ]  and beta catenin  [ **CTNNB1** ]  is extremely valuable for the diagnosis of metastatic occurrence.British Journal of Cancer 2003 8 57 563.  Ref: 12888830 Br J Cancer, 2003
- Differential expression of E cadherin  [ **CDH1** ]  and beta catenin  [ **CTNNB1** ]  in primary and metastatic Wilms s tumours.  Ref: 12890743 Mol Pathol, 2003
- In the metastatic process, reduction in cell to cell adhesion including E cadherin  [ **CDH1** ]  catenin  [ **CTNNB1** ]  cell adhesion complex is an essential step.  Ref: 12888830 Br J Cancer, 2003
